# Estimation of a general time-dependent Hamiltonian for a single qubit

**DOI:** 10.1038/ncomms11218

**Published:** 2016-04-14

**Authors:** L. E. de Clercq, R. Oswald, C. Flühmann, B. Keitch, D. Kienzler, H. -Y. Lo, M. Marinelli, D. Nadlinger, V. Negnevitsky, J. P. Home

**Affiliations:** 1 Institute for Quantum Electronics, ETH Zürich, Otto-Stern-Weg 1, 8093 Zürich, Switzerland

## Abstract

The Hamiltonian of a closed quantum system governs its complete time evolution. While Hamiltonians with time-variation in a single basis can be recovered using a variety of methods, for more general Hamiltonians the presence of non-commuting terms complicates the reconstruction. Here using a single trapped ion, we propose and experimentally demonstrate a method for estimating a time-dependent Hamiltonian of a single qubit. We measure the time evolution of the qubit in a fixed basis as a function of a time-independent offset term added to the Hamiltonian. The initially unknown Hamiltonian arises from transporting an ion through a static laser beam. Hamiltonian estimation allows us to estimate the spatial beam intensity profile and the ion velocity as a function of time. The estimation technique is general enough that it can be applied to other quantum systems, aiding the pursuit of high-operational fidelities in quantum control.

## Abstract (web summary)

Time-varying Hamiltonians can be reconstructed experimentally if the variation takes place in a single basis, but more general cases are complicated. Here, the authors present an approach to estimate the general time-dependent Hamiltonian of a single spin-qubit and apply it to a trapped-ion transported through a static laser beam.

## Graphical abstract

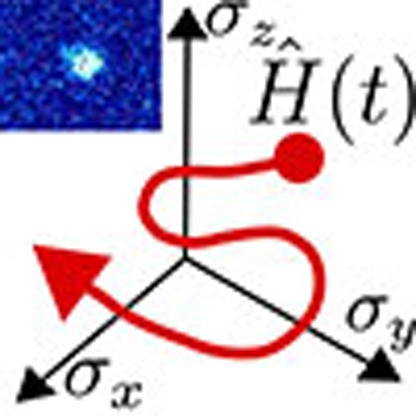

Estimation of the underlying dynamics, which drive the evolution of systems is a key problem in many areas of physics and engineering. This knowledge allows control inputs to be designed, which account for imperfections in the physical implementation. For closed quantum systems, the time dependence of a system is driven by the Hamiltonian through Schrödinger's equation. If the Hamiltonian is static in time, a wide range of techniques have been proposed for recovering the Hamiltonian^1^,^2^,^3^,^4^, which have been applied to a variety of systems including chemical processes^5^ and quantum dots^6^,^7^. These methods often involve estimation of the eigenvectors and eigenvalues of the Hamiltonian via spectroscopy, or through pulse–probe techniques for which a Fourier transform of the time evolution gives information about the spectrum.

These methods are not directly applicable to time-dependent Hamiltonians, which are becoming increasingly important as quantum engineering pursues a combination of high-operational fidelities and speeds, often involving fast variation of control fields, which are particularly susceptible to distortion before reaching the quantum device^8^,^9^,^10^,^11^,^12^. The time-varying case has thus far been studied in cases where the variation is along a single dimension in the Hilbert space, which for the commonly studied spin is a single spatial direction. In the case that the measured fields dominate the evolution (strong field limit), measurement of the system evolution as a function of time suffices for the reconstruction. For fields which are weaker than other available control fields (weak-field limit) the latter can be used to modulate the effect of the signal Hamiltonian on the quantum system^13^,^14^,^15^, providing an excellent signal-to-noise ratio. A further complication arises when a time-varying Hamiltonian contains non-commuting terms (for example, time-variation along two spin axes), because the evolution of the quantum system depends not only on their separate influences, but also on products arising from the non-commutativity. For unspecified time-dependent coefficients, no analytical solution to Schrödinger's equation exists^16^,^17^. In the weak-field limit, strong control fields can be used to separate out the different components using modulation, however, when the Hamiltonian itself is strong (as is the case in fast quantum control) these techniques cannot be applied.

In this article, we propose and demonstrate a method for reconstructing a general time-dependent single qubit Hamiltonian with non-commuting terms. The technique involves observing the evolution of the spin projection on the *z*-axis, while applying a static offset to one of the terms of the Hamiltonian. By varying the static offset, we build up data sets, which contain sufficient information to extract the full time-dependent Hamiltonian. Parameterizing the two time-dependent terms using basis splines (B-splines), we introduce an iterative fitting technique, which finds the Hamiltonian that best matches the data. We benchmark the reconstruction method experimentally by transporting a single trapped ion through a static laser beam, a technique suited to scaling up trapped-ion quantum information processing^18^,^19^. We perform two consistency checks on the Hamiltonian estimation using four separate reconstructions. For the first two, we compare two cases which use the same ion velocity profile, but different laser beam positions. For the second consistency check, we use the same laser beam, but change the velocity profile between the two by using different sets of time-varying control potentials. The method produces consistent experimental parameters in both cases, indicating the success of the reconstruction technique. Our method is applicable to spin Hamiltonians of the general form 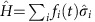, where the *f*_*i*_(*t*) are arbitrary time-dependent functions and 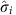 are the Pauli operators.

## Results

### Hamiltonian estimation method

In our experiments, a Hamiltonian with two non-commuting time-dependent terms arises when we perform quantum logic gates by transporting an ion through a static laser beam^18^,^19^. In this case, the Hamiltonian describing the interaction between the ion and the laser can be written in an appropriate rotating frame as


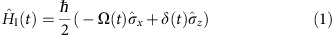


which includes a time-varying Rabi frequency Ω(*t*), and an effective detuning *δ*(*t*), which is related to the first-order Doppler shift of the laser in the rest frame of the moving ion (see Methods for details).

To reconstruct the Hamiltonian, we make use of two additional capabilities. First, we can switch-off the Hamiltonian at time *t*_off_ on a timescale much faster than the qubit evolution. Subsequently measuring in the 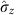 basis, we can obtain 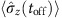. On its own, this does not allow us to separate the contributions from Ω(*t*) and *δ*(*t*). To do this, we use a second capability, which is the ability to add a controlled offset 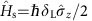 to the Hamiltonian, resulting in 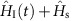. The resulting spin measurement is now dependent on both *t*_off_ and the set value of *δ*_L_. Repeating the experiment for a range of values of *δ*_L_ but with otherwise identical settings, we obtain an estimate of the expectation value which we denote as 
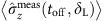
.

Hamiltonian extraction involves finding the functions *δ*(*t*) and Ω(*t*), which generate spin populations 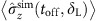 that most closely match the data. We minimize the reduced *χ*^2^ cost function.


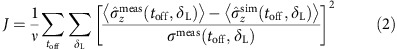


where *ν*=*N*−*n*−1 is the number of degrees of freedom, with *N* the number of data points, *n* the number of fitting parameters and *σ*^meas^(*t*_off_, *δ*_L_) the s.e. on the estimated 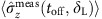. This is subject to the initial condition 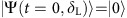, and the following restrictions, which are imposed by quantum mechanics


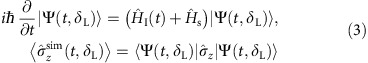


for all *δ*_L_.

One challenge in obtaining an estimate for the Hamiltonian is that we must optimize over continuous functions *δ*(*t*) and Ω(*t*). To address this, we represent *δ*(*t*) and Ω(*t*) with a linear combination of B-spline polynomials, which allow the construction of smooth functions using only a few parameters^20^. Any smooth function *S*(*t*) can be written in terms of B-spline polynomials *B*_*i*,*k*_(*t*) and a set of weights *α*_*i*_ as


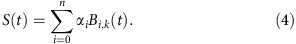


The polynomial B-spline functions *B*_*i*,*k*_(*t*) are of order *k* with each polynomial centred at a time *t*_*i*_, which is parameterized by the index *i*. Further details and an example can be found in Methods. Using the B-spline form for *δ*(*t*) and Ω(*t*), the cost function is minimized by adjusting the weights of the B-spline decomposition. Solving this optimization problem in general is hard, because it is non-linear and non-convex due to the nature of Schrödinger's equation and the use of projective measurements. This produces a non-trivial relation between the weights and the spin populations as discussed in Methods. To overcome this challenge, we have implemented a method which we call ‘Extending the Horizon Estimation' (EHE) in analogy to a well-established technique called ‘Moving Horizon Estimation' (MHE)^21^.

The key idea is that because our measurement data arises from a causal evolution, we can also estimate the Hamiltonian in a causal way. Instead of optimizing *J* over the complete time span at once, we first restrict ourselves to a small, initial time span reaching only up to the start of the qubit dynamics 0<*t*_off_<*T*_0_, where we denote *T*_0_ as the time horizon for the first step. Optimizing *J* over this short time span requires fewer optimization parameters and is simpler than attempting to optimize over the full data set. Once we have solved this small sub-problem, we extend the time horizon to *T*_1_ where 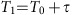 and re-run the optimization, extrapolating the results of the initial time span into the extended region to provide good starting conditions for the subsequent optimization. This procedure is iterated until the time span extends over the whole data set 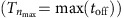. The method allows us to reduce the number of B-spline functions used to represent *δ*(*t*) and Ω(*t*), and also reduces the amount of data considered in the early stages of the fit, when the least is known about the parameters. This facilitates the use of non-linear minimization routines, which are based on local linearization of the problem and converge faster near the optimum. More details regarding the optimization routine can be found in Methods.

Conceptually EHE is very similar to MHE. The main difference is that in MHE the time span has a fixed length and thus its origin is shifted forward in time along with the horizon. In EHE, the origin stays fixed at the expense of having to increase the time span under consideration. MHE avoids this by introducing a so-called arrival cost to approximate the previous costs incurred before the start of the time span. This keeps the computational burden fixed over time, which is very important as MHE is usually used to estimate the state of a system in real-time, often on severely constrained embedded platforms. Since neither constraint applies to our problem, we decided to extend the horizon rather than finding an approximate arrival cost. This is advantageous since finding the arrival cost in the general case is still an open problem^22^. Due to the similarity between MHE and EHE, we anticipate future improvements by adapting techniques used in MHE to EHE. This might be used to reduce the data-processing required for reconstruction, which for EHE scales as 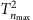.

### Experimental implementation

To test the ability of the method to reliably extract a Hamiltonian from data, we apply it to the Hamiltonian for an trapped-ion qubit during transport through a near-resonant laser beam. Our qubit is encoded in the electronic states of a calcium ion, which is defined by 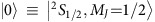 and 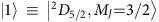. This transition is well-resolved from all other transitions, and has an optical frequency of *ω*_0_/(2*π*)≃411.0420 THz. The laser beam points at 45° to the transport axis, and has an approximately Gaussian spatial intensity distribution. The time-dependent velocity 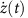 of the ion is controlled by adiabatic translation of the potential well in which the ion is trapped. This is implemented by applying time-varying potentials to multiple electrodes of a segmented ion trap, which are generated using a multi-channel arbitrary-waveform generator, each output of which is connected to a pair of electrodes via a passive third-order low-pass Butterworth filter. The result is that the ion experiences a time-varying Rabi frequency Ω(*t*) and a laser phase which varies with time as Φ(*t*)=*φ*(*z*(*t*))−*ω*_L_*t*, where *φ*(*z*(*t*))=*k*_*z*_(*z*(*t*))*z*(*t*) with *k*_*z*_(*z*(*t*)) the laser wave vector projected onto the transport axis at position *z*(*t*) and *ω*_L_ the laser frequency. The spatial variation of *k*_*z*_(*z*(*t*)) accounts for the curvature of the wavefronts of the Gaussian laser beam. To create a Hamiltonian of the form of equation (1), we work with the differential of the phase, which gives a detuning 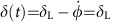

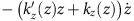
 with *δ*_L_=*ω*_L_−*ω*_0_ the laser detuning from resonance. For planar wavefronts, 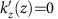, and *δ*(*t*) corresponds to the familiar expression for the first-order Doppler shift (see Methods for details).

**Figure 1:**
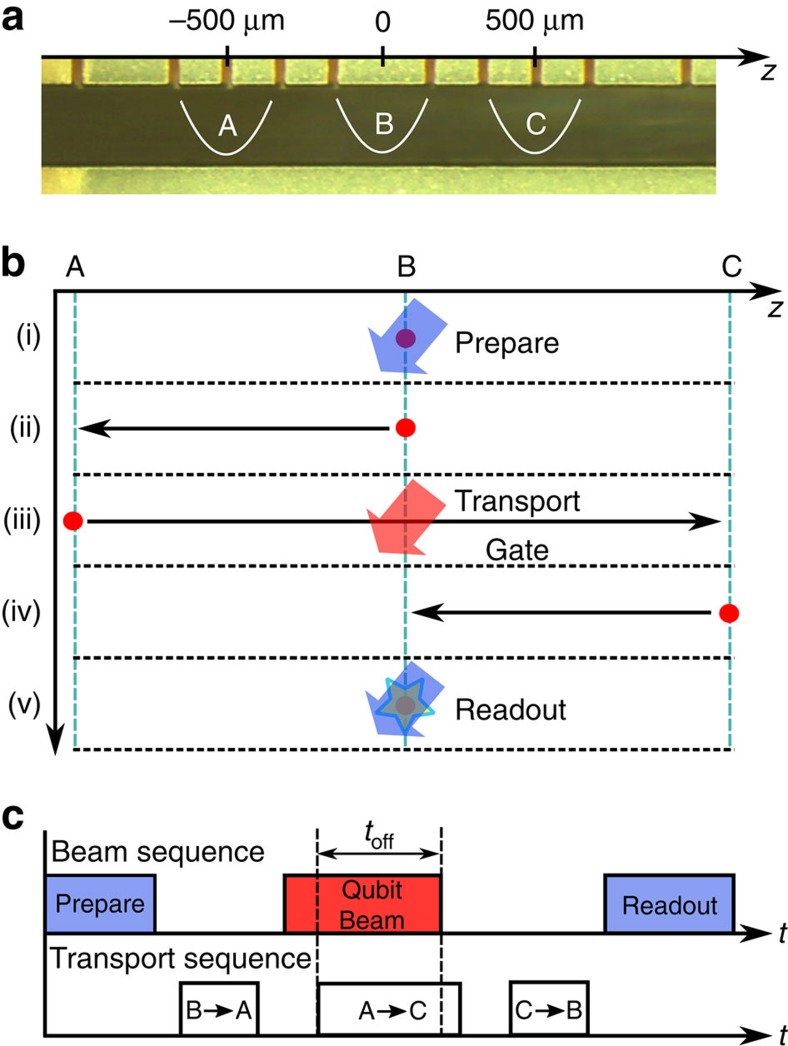
Experimental sequence and timing. (**a**) The experiment is carried out in three zones of the trap indicated by A, B and C. (**b**) The experimental sequence involves steps (i) through (v). Preparation and readout are carried out on the static ion in zone B. The qubit evolves while the ion is transported through the laser beam in zone B in a transport operation taking the ion from zone A to zone C. (**c**) Experimental sequence showing the timing of applied laser beams and ion transport, including shutting off the laser beam during transport.

The experimental sequence is depicted in Fig. 1. We start in zone B by cooling all motional modes of the ion to 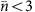 using a combination of Doppler and electromagnetically induced transparency cooling^23^, and then initialize the internal state by optical pumping into 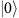. The ion is then transported to zone A, and the laser beam used to implement the Hamiltonian is turned on in zone B. The ion is then transported through this laser beam to zone C. During the passage through the laser beam, we rapidly turn the beam off at time *t*_off_ and thus stop the qubit dynamics. The ion is then returned to the central zone B to perform state readout, which measures the qubit in the computational basis 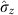 (for more details see Methods). The additional Hamiltonian 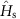 is implemented by offsetting the laser frequency used in the experiment by a detuning *δ*_L_. For each setting of *t*_off_ and *δ*_L_ the experiment is repeated 100 times, allowing us to obtain an estimate for the qubit populations 
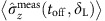
.

**Figure 2:**
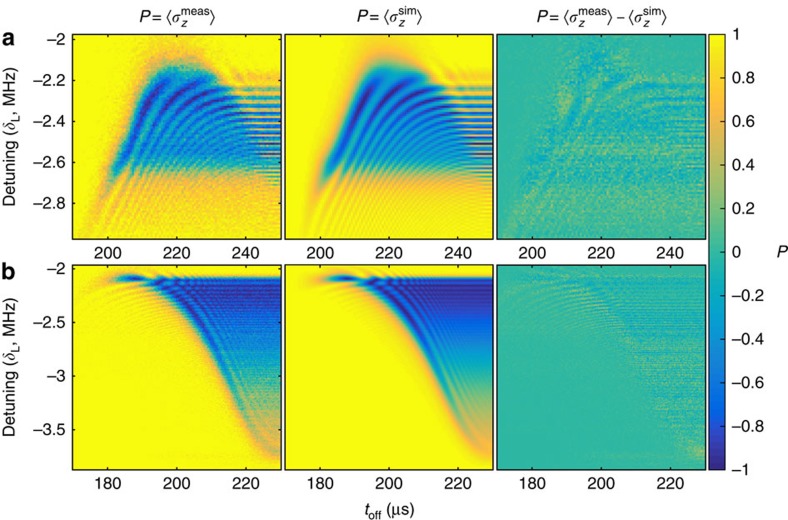
Measured data, best fit and residuals. Spin population as a function of detuning and switch-off time of the laser beam. **a** is for a laser beam centred in zone B, while for **b** the beam was displaced towards zone C by 64 *μ*m. From left to right are plots of the experimental data, the populations generated from the best fit Hamiltonian, and the residuals. Each data point results from 100 repetitions of the experimental sequence. The data in **a** consist of an array of 100 × 101 experimental settings, while that shown in **b** consists of an array of 201 × 201 settings. This leads to smaller error bars in the reconstructed Hamiltonian for the latter. For the Hamiltonian estimation, the data was weighted according to quantum projection noise.

We first perform a comparison in which the ion velocity is the same but the beam position is changed. Thus we expect to obtain two different profiles for Ω(*t*) but the same velocity profile, which is closely related to *δ*(*t*). Experimental data is shown in Fig. 2 alongside the results of fitting performed using our iterative method. The beam positions used for each data set differ by ∼64 μm along the transport axis, but the transport waveform used was identical. It can be seen from the residuals that the estimation is able to find a Hamiltonian, which results in a close match to the data.

**Figure 3:**
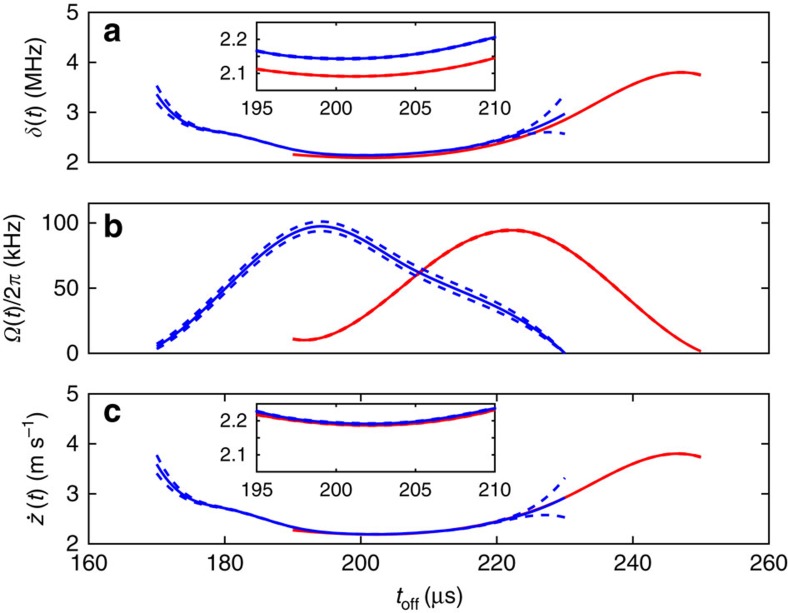
Estimates of time-dependent co-efficients. (**a**) The effective detuning *δ*(*t*) and (**b**) Rabi frequency Ω(*t*) obtained from the two data sets. Blue and red solid lines show data obtained having the beam centred in zone B and with the beam displaced by a few tens of microns. Dashed lines indicate the s.e. on the mean of these estimates, which are obtained using resampling. For **a** the inset shows a close-up of the estimated *δ*(*t*) in the regions where the estimates overlap, showing that these do not give the same value. (**c**) The estimated velocity 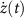 of the ion obtained after applying wavefront correction. The inset shows that this produces consistent results.

The estimated coefficients of the Hamiltonian extracted from the two data sets are shown in Fig. 3a,b. To estimate the relevant errors of our reconstruction, we have performed non-parametric resampling with replacement, optimizing for the solution using the same set of B-spline functions as was used for the experimental data to provide a new estimate for the Hamiltonian. This is repeated for a large number of samples, resulting in a distribution for the estimated values of *δ*(*t*) and Ω(*t*) from which we extract statistical properties such as the s.e. The error bounds shown in Fig. 3 correspond to the s.e. on the mean obtained from these distributions (see Methods for further details). It can be seen that the values of *δ*(*t*) for the two different beam positions have a similar form but a fixed offset for the region where the reconstructions overlap. We believe that this effect arises from the non-planar wavefronts of the laser beam. Inverting the expression for *δ*(*t*) to obtain the velocity of the ion, we find 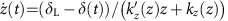. Using this correction, we find that the two velocity profiles agree if we assume that the ion passes through the centre of the beam at a distance of 2.27 mm before the minimum beam waist, a value which is consistent with experimental uncertainties due to beam propagation and possible mis-positioning of the ion trap with respect to the fixed final focusing lens. The velocity estimates taking account of this effect are shown in Fig. 3c.

**Figure 4:**
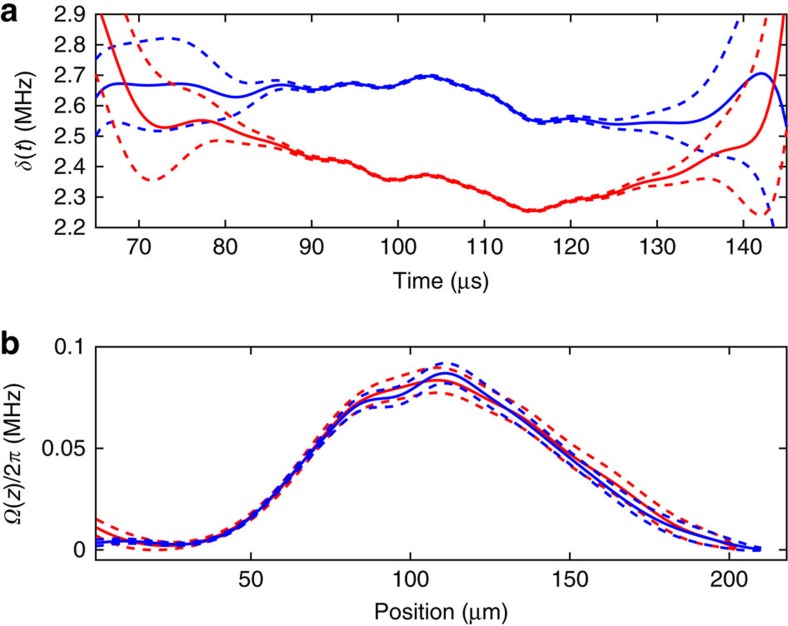
Time-dependent detuning and spatial Rabi frequency. (**a**) The estimated *δ*(*t*) obtained from the second pair of data sets (Fig. 8 in Methods). (**b**) The estimated Rabi frequency Ω(*t*) for the same two data sets. In each part, the blue and red solid lines show data obtained using different velocity profiles. Dashed lines indicate the s.e. on the mean of these estimates, which are obtained using resampling.

Our second comparison involves using two different velocity profiles but with a common beam position. The resolution in both time and detuning were lower in this case than for the data shown in Fig. 2 (see Methods for the data). Figure 4 shows the results of the reconstruction. We observe that the estimated Rabi frequency profiles agree to within the error bars of the reconstruction. One interesting feature of this plot is that the error bars produced from the resampled data sets increase near the peak. We believe that this happens because the sampling time of the data is 0.5 μs, which starts to become comparable to the Rabi frequency (the Nyquist frequency is 1 MHz). To optimize the efficiency of our method, it would be advantageous to run the reconstruction method in parallel with data taking, thus allowing updating of the sampling time and frequency resolution based on the current estimates of parameter values.

## Discussion

Our method for directly obtaining a non-commuting time-dependent Hamiltonian uses straightforward measurements of the qubit state in a fixed basis as a function of time and a controlled offset to the Hamiltonian. Unlike schemes based on dynamical modulation or continuous strong driving, it avoids the need for control fields which act more strongly on the qubit than the Hamiltonian to be measured. This is a key advantage in quantum technologies where the Hamiltonian of interest is often already close to the limit of system drive strength. A process-tomography-based approach would require that for every time step multiple input states be introduced, and a measurement made in multiple bases^24^,^25^,^26^. This requires a much greater level of control than the method presented above. An effective modulation of the measurement basis arises in our approach due to the additional detuning *δ*_L_. Nevertheless, it is also worth noting that tomography provides more information than our method: it makes no assumptions about the dynamics aside from that of a completely positive map while we require coherent dynamics. Extensions to our work are required to provide a rigorous estimation of the efficiency of the method in terms of the precision obtained for a given number of measurements, and to see whether a similar approach could be taken for non-unitary dynamics. We have recently used these methods to improve the control over the ion velocity, which is of direct value in optimizing transport operations in scalable trapped-ion quantum information processing^11^,^12^,^27^, and will be essential for realizing multi-qubit transport gates^18^. We expect them to be applicable across a wide range of physical systems where such control is available, including those considered for quantum computation^4^,^6^,^7^,^28^,^29^,^30^,^31^.

## Methods

### Derivation of Hamiltonian

The interaction of a laser beam with frequency *ω*_L_ and wave vector **k**(**z**(*t*)) with a two-level atom with resonant frequency *ω*_0_ and time-dependent ion position **z**(*t*)=(0, 0, *z*(*t*)) can be described in the Schrödinger picture by the Hamiltonian


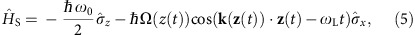


where the Rabi frequency Ω(*z*(*t*)) gives the interaction strength between the laser and the two energy levels. We can define the laser phase at the position of the ion as Φ(*t*)=*φ*(*t*)−*ω*_L_*t* with *φ*(*t*)=**k**(**z**(*t*))·**z**(*t*)=*k*_*z*_(*z*(*t*))*z*(*t*) and *k*_*z*_(*z*(*t*))=|**k**|cos(*θ*(*t*)) being the projection of the laser beam onto the *z*-axis along which the ion is transported. Here *θ*(*t*) is the angle between the wave vector **k**(*z*(*t*)) and the transport axis evaluated at position *z*(*t*). Moving to a rotating frame using the unitary transformation 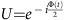 and applying the rotating wave approximation with respect to optical frequencies, we obtain


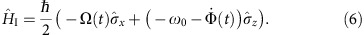


Defining a static detuning *δ*_L_=*ω*_L_−*ω*_0_, we obtain


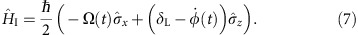


with


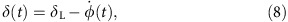


which is the expression used in the main text.

### B-spline curves and optimization algorithm

The set of polynomial B-spline functions *B*_*i*,*k*_(*t*) of order *k* are recursively defined over the index *i* over a set of points **K**={*t*_0_, *t*_1_, .., *t*_*n*+*k*_}, which is referred to as the knot vector^20^.


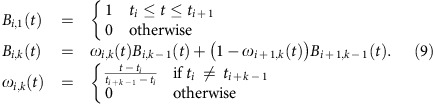


**Figure 5:**
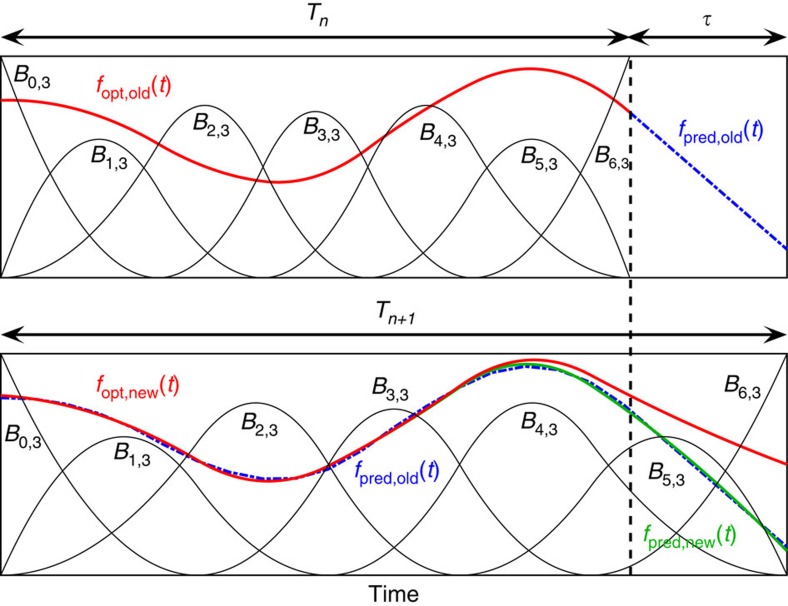
Extending the horizon estimation. The steps performed when extending the time horizon from *T*_*n*_ to *T*_*n*+1_ are illustrated. We first predict in the old basis, then move to the new basis, and finally optimize again. The figure also shows the B-splines *B*_*i*,*k*_(*t*).

Figure 5 gives a visualization of the B-splines *B*_*i*,*k*_(*t*) and a B-spline curve. The B-spline construction ensures that any linear combination of the B-splines is continuous and has (*k*−2) continuous derivatives. The knot vector **K** determines how the basis functions are positioned within the interval [*t*_0_, *t*_*n*+*k*_]. We notice that for our Hamiltonian the spacing of the B-splines is not critical, which we think is due to the smoothness of the variations in our Hamiltonian parameters *δ*(*t*) and Ω(*t*). We therefore used the Matlab function spap2 to automatically choose a suitable knot vector and restricted ourselves to optimizing the coefficients *α*_*i*_. We collect all coefficients *α*_*i*_ for *δ*(*t*) and Ω(*t*) and store them in a single vector ***α***.

A detailed algorithmic summary of our implementation of the EHE method is given below.

1. Searching for a starting point: here we reconstruct the Hamiltonian for a first, minimal time horizon such that we can then use this as a starting point to iteratively extend the horizon as described in step 2.

This procedure is used to provide a starting point for the optimization over the initially chosen window, which is typically performed with a set of higher order B-splines. From this starting point, we iteratively extend the fitting method to the full data set as follows.

1. Extend the horizon: this step is repeated until the whole time horizon is covered. It consists of the following sequence, which is illustrated in Fig. 5.

If all these fail, we have to resort to increasing the bound on 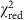.

1. Post-processing: the following steps are optional and were performed manually in cases where we wished to improve the fit or examine its behaviour.

### Wavefront correction

For plane waves, we find that 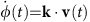, which is the well-known expression for the first-order Doppler shift. For transport through a real Gaussian beam, the wave vector direction changes with position. Taking this into account, the derivative of *φ*(*t*) becomes


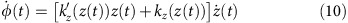


where 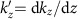 and 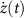 is the component of the ion velocity along the *z*-axis. We extract *δ*(*t*) using our Hamiltonian estimation procedure, thus to obtain the velocity of the ion we use


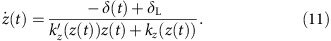


**Figure 6:**
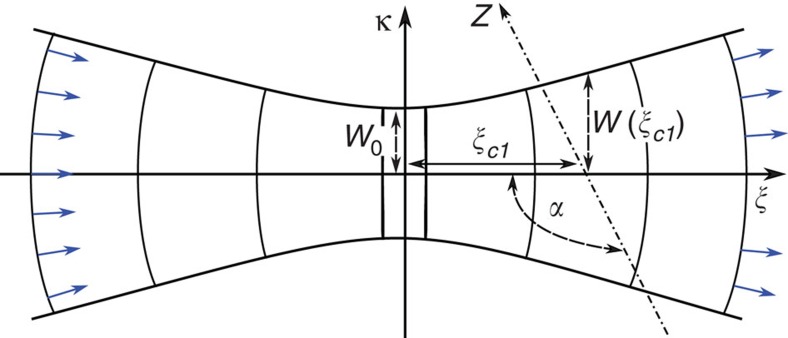
Beam and ion transport. The beam propagation direction lies along the *ξ*-axis and the ion is transported along the *z*-axis lying on the *κξ*-plane as indicated. Normalized vectors representing **e**_*l*_(*κ*, *ξ*) lying perpendicular to the wavefronts are indicated by the blue arrows.

As the ion moves through the beam it experiences the same magnitude of the wave vector |**k**|=2*π*/*λ*, but the angle *θ* between the ion direction and the wave vector changes. Written as a function of this angle, the velocity becomes


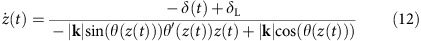


where *θ*′(*z*(*t*))=d*θ*(*z*(*t*))/d*z*(*t*). We parameterize our Gaussian beam according to Fig. 6. The phase is given as a function of both the position along the beam axis *ξ* and the perpendicular distance from this axis *κ* by^32^


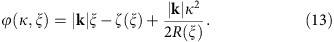


where the Gaussian beam parameters include the beam waist *W*(*ξ*), the radius of curvature *R*(*ξ*), the Rayleigh range *ξ*_R_ and the Guoy phase shift *ζ*(*ξ*). These are given by the expressions


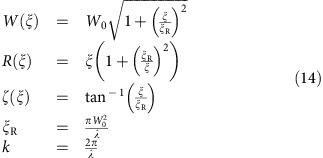


where *W*_0_ is the minimum beam waist and *λ* the laser wavelength. The ion moves along the *z*-axis as shown in Fig. 6. In the *κξ*-plane a unit vector **e**_*l*_(*κ*, *ξ*) perpendicular to the wavefronts is given by


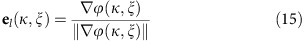


and the unit vector **e**_*v*_ pointing along the direction of transport is given by


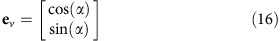


The angle *θ*(*ξ*) between the wave and position vector is then given by the dot product


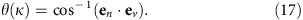


which can be written in terms of the full set of parameters above as


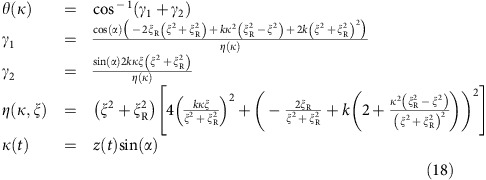


where in our experiments *α*=3*π*/4.

Using equations (12) and (18), we examined the value of *ξ*_cl_ required for the velocity to match for our two beam positions. We find that they agree for *ξ*_cl_=−2.27 mm, which is within the experimental uncertainties for our set-up.

### Error estimation

To estimate the errors of the time-dependent functions, we use non-parametric bootstrapping^33^. The process is summarized as follows:

1. Estimate initial solution: estimate the time-dependent functions from the original data using Hamiltonian estimation.
2. Resampling: create *N*_s_ sample solutions for all time-dependent functions in the following way:
3. Post-process samples:
4. Obtain statistics:

In evaluating errors using bootstrapping, we use the same set of spline polynomials as were used for the final optimization stage in the data, which makes the reconstruction more reliable in converging to a minimum. We thus expect that the parameter space explored in evaluating the errors is not the same as for the *ab initio* estimation of the Hamiltonian. This is apparent in the regions of the data where the final estimate has large error bounds (for example, in Fig. 4), where an even larger spread might be expected (the dynamics has stopped evolving at this point). We think that the net effect is to under-estimate the errors in the regions where the Hamiltonian is uncertain, but that the error bars given in the central region (where the Hamiltonian is well-defined) are close to what would be obtained through a full optimization.

**Figure 7:**
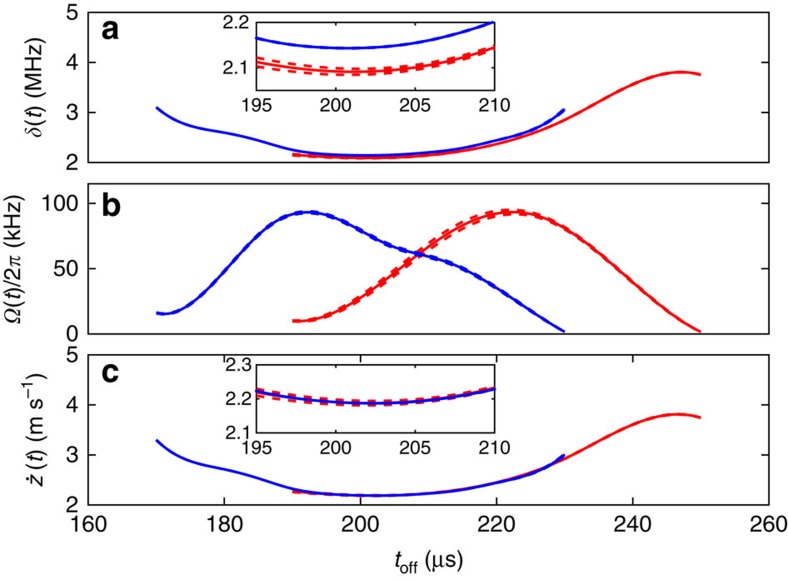
Parametric bootstrap resampling. Predictions for the effective detuning *δ*(t) in **a**, Rabi frequency Ω(*t*) in **b** and velocity 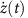 in **c**. Blue and red solid lines show data obtained having the beam centred in zone B and with the beam displaced by a few tens of micron. Dashed lines represent the s.e. on the mean of these estimates obtained using parametric bootstrap resampling, assuming quantum projection noise. This can be compared with the error bounds obtained from the non-parametric method, which are shown in Fig. 3 in the main text. The bounds are tighter for the parametric bootstrapping.

We have also applied parametric bootstrapping to obtain the error bounds shown in Fig. 7. The difference to the non-parametric case is that in point (2) the samples are created using the solutions obtained from (1) and adding quantum projection noise. For each sample the Hamiltonian is estimated. The estimates from multiple samples are used to construct error bounds in the same manner as for the non-parametric resampling. We have found that the error bounds obtained from parametric bootstrapping are lower compared with that of the non-parametric case as shown in Fig. 3. We think this is due to the latter exploring deviations around a single minimum in the optimization landscape, while the case resampling arrives at different local minima, which are spread over a wider region.

### Single beam profile with two different velocity profiles

**Figure 8:**
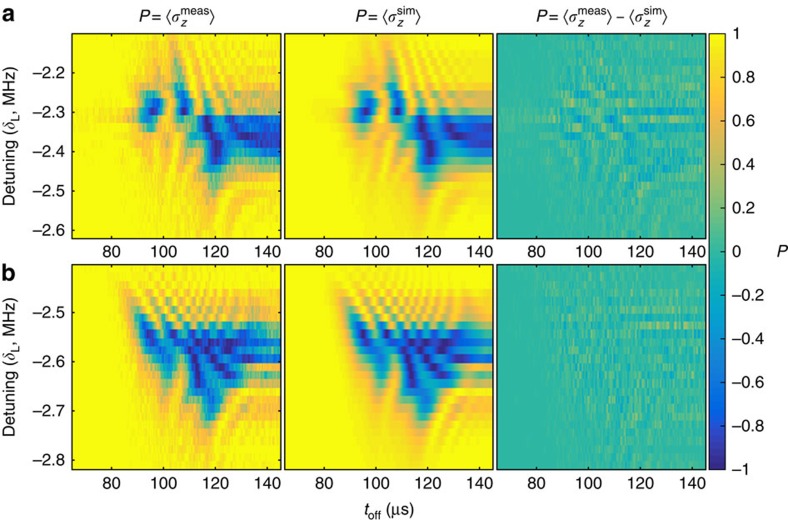
Measured data, estimation and residuals. Spin population as a function of detuning and switch-off time of the laser beam, for the data sets used to obtain the reconstructed parameters shown in Fig. 4. **a** corresponds to the blue reconstruction while **b** to the red one. Each data point results from 100 repetitions of the experimental sequence. For the Hamiltonian estimation, the data was weighted according to quantum projection noise.

To verify that our method can also consistently estimate the Rabi frequency profile, we measure a second pair of data sets in which we take two different velocity profiles using the same beam position. This data is shown in Fig. 8. Also shown are the best-fits obtained from the reconstructed Hamiltonians. The parameter variations obtained from the reconstructed Hamiltonians for these data sets can be found in the main text in Fig. 4. The sampling rate of the data in these data sets was 2 MHz, resulting in a Nyquist frequency of 1 MHz.

## Additional information

**How to cite this article:** de Clercq, L. E. *et al*. Estimation of a general time-dependent Hamiltonian for a single qubit. *Nat. Commun.* 7:11218 doi: 10.1038/ncomms11218 (2016).
